# High Throughput Transcriptome Data Analysis and Computational Verification Reveal Immunotherapy Biomarkers of Compound Kushen Injection for Treating Triple-Negative Breast Cancer

**DOI:** 10.3389/fonc.2021.747300

**Published:** 2021-09-17

**Authors:** Xinkui Liu, Yang Wu, Yingying Zhang, Dechao Bu, Chao Wu, Shan Lu, Zhihong Huang, Yurong Song, Yi Zhao, Fengying Guo, Peizhi Ye, Changgeng Fu, Liangliang Shen, Jingyuan Zhang, Haojia Wang, Xianchun Duan, Jiarui Wu

**Affiliations:** ^1^School of Chinese Pharmacy, Beijing University of Chinese Medicine, Beijing, China; ^2^Key Laboratory of Intelligent Information Processing, Advanced Computer Research Center, Institute of Computing Technology, Chinese Academy of Sciences, Beijing, China; ^3^Department of Vascular Neurosurgery, New Era Stroke Care and Research Institute, The People’s Liberation Army (PLA) Rocket Force Characteristic Medical Center, Beijing, China; ^4^Pervasive Computing Research Center, Institute of Computing Technology, Chinese Academy of Sciences, Beijing, China; ^5^School of Traditional Chinese Medicine, Beijing University of Chinese Medicine, Beijing, China; ^6^School of Management, Beijing University of Chinese Medicine, Beijing, China; ^7^National Cancer Center/National Clinical Research Center for Cancer/Cancer Hospital, Chinese Academy of Medical Sciences and Peking Union Medical College, Beijing, China; ^8^Xiyuan Hospital, China Academy of Chinese Medical Sciences, Beijing, China; ^9^Department of Pharmacy, The First Affiliated Hospital of Anhui University of Traditional Chinese Medicine, Hefei, China

**Keywords:** compound kushen injection, triple-negative breast cancer, RNA sequencing, tumor microenvironment, prognostic signature

## Abstract

**Background:**

Although notable therapeutic and prognostic benefits of compound kushen injection (CKI) have been found when it was used alone or in combination with chemotherapy or radiotherapy for triple-negative breast cancer (TNBC) treatment, the effects of CKI on TNBC microenvironment remain largely unclear. This study aims to construct and validate a predictive immunotherapy signature of CKI on TNBC.

**Methods:**

The UPLC-Q-TOF-MS technology was firstly used to investigate major constituents of CKI. RNA sequencing data of CKI-perturbed TNBC cells were analyzed to detect differential expression genes (DEGs), and the GSVA algorithm was applied to explore significantly changed pathways regulated by CKI. Additionally, the ssGSEA algorithm was used to quantify immune cell abundance in TNBC patients, and these patients were classified into distinct immune infiltration subgroups by unsupervised clustering. Then, prognosis-related genes were screened from DEGs among these subgroups and were further overlapped with the DEGs regulated by CKI. Finally, a predictive immunotherapy signature of CKI on TNBC was constructed based on the LASSO regression algorithm to predict mortality risks of TNBC patients, and the signature was also validated in another TNBC cohort.

**Results:**

Twenty-three chemical components in CKI were identified by UPLC-Q-TOF-MS analysis. A total of 3692 DEGs were detected in CKI-treated versus control groups, and CKI significantly activated biological processes associated with activation of T, natural killer and natural killer T cells. Three immune cell infiltration subgroups with 1593 DEGs were identified in TNBC patients. Then, two genes that can be down-regulated by CKI with hazard ratio (HR) > 1 and 26 genes that can be up-regulated by CKI with HR < 1 were selected as key immune- and prognosis-related genes regulated by CKI. Lastly, a five-gene prognostic signature comprising two risky genes (MARVELD2 and DYNC2I2) that can be down-regulated by CKI and three protective genes (RASSF2, FERMT3 and RASSF5) that can be up-regulated by CKI was developed, and it showed a good performance in both training and test sets.

**Conclusions:**

This study proposes a predictive immunotherapy signature of CKI on TNBC, which would provide more evidence for survival prediction and treatment guidance in TNBC as well as a paradigm for exploring immunotherapy biomarkers in compound medicines.

## Introduction

Triple-negative breast cancer (TNBC) is the most malignant and aggressive subtype of breast cancer, which is pathologically featured by the lack of estrogen receptor (ER), progesterone receptor (PR), and human epidermal growth factor receptor 2 (HER2) expression (1–3). Despite its clinical characteristics of high invasion, metastasis, a high rate of early relapse, a dismal prognosis, and a limited response to conventional chemotherapies or targeted therapies, immunotherapy are showing great promise and its use has been approved in combination with traditional treatment options in TNBC (2, 4–9).

Compound kushen injection (CKI) is an anticancer Chinese patent medicine (CPM) approved by National Medical Products Administration (NMPA) in China, which is extracted from the roots of two medical herbs Kushen (*Radix Sophorae Flavescentis*) and Baituling (*Rhizoma Heterosmilacis*) *via* standardized Good Manufacturing Practice (GMP) (10, 11). Multiple bioactive ingredients in compound kushen injection have been extensively reported, such as matrine, oxymatrine, oxysophocarpine and sophocarpine (11–13). CKI has been widely used alone or in combination with chemotherapy or radiotherapy in the treatment of patients with liver cancer, lung cancer, breast cancer, gastric cancer, colorectal cancer and other cancer types (12–19), indicating that it has a broad spectrum of anti-cancer activity. Notably, increasing clinical evidence has shown that CKI synergizes the efficacy of chemotherapy and radiotherapy, decreases the toxicity or side effects induced by chemotherapy and radiotherapy, enhances quality of life, and improves the immune function of cancer patients (14–16). A survey on the use of anti-cancer CPMs among 51,382 insured cancer patients demonstrates that CKI is the second frequently used anti-cancer CPMs and is also the CPM with the highest use rate in 17 cancers; moreover, CKI is also the second commonly used anti-cancer CPMs in breast cancer (20). For breast cancer, a meta-analysis of randomized controlled trials included 16 studies with 1,315 participants reports that CKI combined with chemotherapy might enhance performance status and reduce the rate of adverse drug reactions among postoperative patients with breast cancer (16). Meanwhile, CKI has been found to inhibit human breast cancer stem-like cells by inactivating the canonical Wnt/β-catenin pathway (12). Furthermore, a recent study aimed at illustrating the effect of CKI on tumor immunity demonstrates that CKI relieves the immunosuppression mediated by tumor-associated macrophages and afterwards alleviates the immunosuppressive effects on CD8^+^ T cells, which enhances the efficacy of low-dose sorafenib and avoids chemotherapy-induced adverse effects (10). However, the effects and underlying regulatory mechanisms of CKI on TNBC microenvironment are still largely unclear.

The tumor microenvironment (TME) plays a crucial role in tumor initiation, progression, relapse, metastasis and treatment response (21, 22). For example, tumor-infiltrating lymphocytes (TILs) in the tumor and stroma have shown an important prognostic value in TNBC, and more importantly TILs have also been identified as an indicator of response not only to neoadjuvant or adjuvant chemotherapy but also to anti-PD-1 or anti-PD-L1 antibodies in patients with TNBC (23–29). Immunohistochemistry (IHC), immunofluorescence (IF), flow cytometry and cytometry by time of flight (CyTOF) mass spectrometry as traditional techniques are commonly used to quantify cells from the complex components in TME (30). However, these methods have the weaknesses of laborious, low throughput and demanding preselected cell markers, while single-cell RNA sequencing (scRNA-seq) is expensive to be used on large patient cohorts and demands particular sample preparation at this stage, which hinder their application in a large number of clinical samples (30). Fortunately, multiple computational approaches have been developed to estimate the relative abundance of distinct cell types in the TME based on bulk expression data, which provide a systematic strategy to comprehensively explore the TME in an unbiased manner and most importantly can be applied to existing datasets with thousands of genetically profiled and clinically well-annotated tumor samples (30, 31). Therefore, researchers have developed a large number of signatures based on tumor-infiltrating cells using computational methods in breast cancer, which has provided clinicians with more precise information for deeply understanding the immunogenomic profile of breast cancer, stratifying patients, and predicting patient outcome or treatment response (32–34). Due to the high levels of heterogeneity and complexity in TNBC microenvironment, it remains necessary to propose novel prognostic signatures based on TME-relevant genes in TNBC.

In this study, we detected differential expression genes (DEGs) after analyzing RNA-seq data of CKI on TNBC cells, and later applied the GSVA algorithm to explore significantly changed pathways regulated by CKI. Then, we estimated the relative quantitative infiltration levels of 28 immune cell signatures in TNBC patients with the single sample gene set enrichment analysis (ssGSEA) algorithm. Meanwhile, we divided these patients into different immune cell infiltration patterns with the consensus clustering method and next detected DEGs among these subgroups. Furthermore, we performed univariate Cox analysis to select prognosis-related genes from immune-related genes, and overlapped them with DEGs regulated by CKI. Finally, we constructed a prognostic signature of immune-related genes with the least absolute shrinkage and selection operator (LASSO) regression method to predict mortality risks in TNBC patients, and we also confirmed the predictive capability of this immune gene signature in another TNBC cohort.

## Materials and Methods

### Ultra-Performance Liquid Chromatography Coupled to Quadrupole Time-of-Flight Mass Spectrometry Analysis

CKI (Batch No: 20181034, total alkaloid concentration of 25 mg/mL) was provided by Zhendong Pharmaceutical Co., Ltd (China). The CKI was diluted ten-fold in ultrapure water, and 2 µl of the solution was used for further analysis. Five control compounds were obtained, including sophocarpine (Batch No: 20052711, purity ≥ 99.84%), oxymatrine (Batch No: 20041315, purity ≥ 98.76%), matrine (Batch No: 20200820, purity ≥ 98%), hesperidin (Batch No: 200621, purity ≥ 98%) and sophoridine (Batch No: 19113001, purity ≥ 96.97%). Matrine was purchased from Beijing North Weiye Metrology Institute Co., Ltd (Beijing, China), and the other four compounds were purchased from Beina Biotechnology Institute Co., Ltd (Beijing, China).

The CKI was separated by applying a Waters ACQUITY UPLC BEH C18 column (2.1 mm × 100 mm, 1.7 µm, Waters Corporation, Milford, MA, United States) at 35°C. Mobile phases comprised 0.1% aqueous formic acid and acetonitrile. A gradient elution with the flow rate of 0.2 mL/min was executed as follows: 6% acetonitrile at 0-2 min, 6-15% acetonitrile from 2 to 4 min, 15-25% acetonitrile from 4 to 8 min, 25-45% acetonitrile from 8 to 14 min, 45-60% acetonitrile from 14 to 16 min, 60% acetonitrile from 16 to 18 min, 60-6% acetonitrile from 18 to 19 min, and 6% acetonitrile from 19 to 22 min. The MS analysis was performed using the electrospray ionization (ESI) source in both positive and negative ion modes, and leucine enkephalin was utilized for mass accuracy correction. The temperatures of ion source and desolvation gas were set at 120°C and 350°C, respectively. The flow rates of the cone and the desolvation gas were 50 L/h and 600 L/h, respectively. The capillary voltages were set to 3.0 kV and 2.5 kV in positive and negative ion modes, respectively. The cone and extraction cone voltages were set to 40kV. MS/MS analysis was performed with a low collision energy of 4 eV and a high collision energy of 20-35 eV. The scan area was set at m/z 50-1200. Data acquisition and analysis were conducted with MassLynx™ v4.1 (Waters Co., Ltd) and UNIFI R Scientific Information System v1.7 (Waters Co., Ltd).

### Transcriptome Data Acquisition and Processing

The RNA-seq dataset of CKI on breast cancer MDA-MB-231 cells was download from European Nucleotide Archive (35) with the accession number PRJNA517432 (36, 37), in which 12 samples at 48-hour (three untreated in batch 1, three untreated in batch 2, three CKI-treated in batch 1 and three CKI-treated in batch 2) were included in our study (**Supplementary Table 1**). FastQC (version 0.11.9, Babraham Bioinformatics) was used to check the quality of raw reads before proceeding with downstream analysis. Trim_galore (version 0.6.6, Babraham Bioinformatics) was used to trim adaptors and low-quality sequences. STAR (version 2.7.7a) (38) was then applied to construct a reference genome index based on the DNA sequence and gene transfer format (GTF) files of the reference genome [GRCh38, Ensembl Release 103 (39)], and thereby the trimmed reads were further aligned to the above index by the STAR software and Binary Alignment/Map (BAM) format files were sorted by samtools (version 1.10) (40). Then, read counts data was generated after preparing reference sequences (reference genome: GRCh38, Ensembl Release 103) and calculating expression values by RSEM (v1.3.3) (41).

Differential expression analysis was performed by the DESeq2 (42), edgeR (43) and limma (44, 45) packages, respectively. The official pipelines of the three R packages that consider batch effects in RNA sequencing data in Bioconductor (46) were referenced in our study. The “filterByExpr” function in edgeR was used to screen genes with sufficiently large counts for a statistical analysis and scaling factors were calculated with the trimmed mean of M values (TMM) method (47) to convert raw library sizes into effective library sizes. The apeglm method (48) in the “lfcShrink” function was used to shrink log2 fold changes (FCs) when applying the DESeq2 method to perform differential expression analysis. The significance threshold for differential gene expression screening was set as adjusted *P* < 0.05 and |log_2_FC| > log_2_1.5, and the overlapping genes from the three methods were considered as DEGs. Normalized expression values of RNA-seq data were obtained with the voom algorithm (49), and batch effects were removed by the sva package (50) for further analysis.

### Gene Set Variation Analysis

Pathway analyses were performed on the 50 hallmark gene sets described in the Molecular Signatures Database (MSigDB, version 7.4) (51), and were then accomplished on biological process signatures in the Gene Ontology (GO) (52) and six kind of pathways comprising metabolism, genetic information processing, environmental information processing, cellular processes, organismal systems and human diseases deposited in the Kyoto Encyclopedia of Genes and Genomes (KEGG) database (Last updated: May 1, 2021) (53). The gene sets with at least two genes found in the RNA-seq data were retained, and the GSVA algorithm was called from within the GSVA package (54) to calculate the enrichment score of each biological pathway in each sample with transcriptomic data. Subsequently, the empirical Bayesian approach within the limma package was applied to determine significantly changed pathways, with adjusted *P* < 0.05 as a significant cutoff criterion.

### TNBC Datasets and Samples

The METABRIC (55) cohort (a total of 1,980 patients, 100% female; including 320 patients with ER-, PR- and HER2- status) of breast cancer patients was included as the training set in our study. Non-primary breast cancer cases or patients without expression profiles were excluded, so 298 primary TNBC patients (100% female, average age = 56 ± 14 years) were reserved for further analysis (**Supplementary Table 2**). The event was defined as died of disease and the patients who were living or died of other causes were censored. Furthermore, another cohort with 107 TNBC patients (56) was included as the test set to externally validate the performance of our survival model (GSE58812; 100% female, average age = 57 ± 13 years), and the metastasis-free survival (MFS) time of these cases was extracted (**Supplementary Table 3**). The CEL format files of this microarray data were downloaded and were normalized with the Robust Multi-array Average (RMA) method in the affy package (57).

### Calculation of Microenvironment Cell Abundance

Feature gene sets of 28 subpopulations of tumor-infiltrating immune cells (58) were referenced, which include 28 immune cell types (13 innate and 15 adaptive immune cells), namely, activated dendritic cells (DCs), CD56bright natural killer (NK) cells, CD56dim natural killer (NK) cells, eosinophils, immature dendritic cells (DCs), macrophages, mast cells, myeloid-derived suppressor cells (MDSCs), monocytes, natural killer (NK) cells, Natural killer T (NKT) cells, neutrophils, plasmacytoid dendritic cells (pDCs), activated B cells, activated CD4^+^ T cells, activated CD8^+^ T cells, CD4^+^ central memory T (Tcm) cells, CD8^+^ central memory T (Tcm) cells, CD4^+^ effector memory T (Tem) cells, CD8^+^ effector memory T (Tem) cells, gamma delta T (γδ T) cells, immature B cells, memory B cells, regulatory T (Treg) cells, T follicular helper (Tfh) cells, type 1 T helper (Th1) cells, type 17 T helper (Th17) cells and type 2 T helper (Th2) cells. In the METABRIC cohort, the ssGSEA algorithm was called from within the GSVA package to quantify the relative infiltration of the 28 immune cell types in the tumor microenvironment (TME). The normalized enrichment score (NES) of these immune cell signatures calculated by ssGSEA was utilized to indicate the relative abundance of each immune cell in each TNBC sample. The prognostic value of each cell subset was evaluated with the univariate Cox proportional hazards model. ESTIMATE (59) was applied to evaluate the infiltration level of immune cells (immune score), the level of stromal cells (stromal score) and tumor purity for each patient.

### Screening of TME- and Prognosis-Related Genes

The unsupervised clustering Pam method based on Euclidean and Ward’s linkage was called from within the ConsensuClusterPlus package (60) to determine the optimal number of stable immune-based TNBC clusters, and was repeated 100,000 times to ensure the stability of classification. The hierarchical clustering and k-means clustering were performed to confirm the robustness of the clustering. Principle component analysis (PCA) and t-Distributed Stochastic Neighbor Embedding (t-SNE) were further applied for dimensional reduction of TNBC patients based on tumor-infiltrating immune cells. Kaplan-Meier survival analysis with the log-rank test was implemented to compare the differences in prognosis among distinct immune subgroups. To identify TME-relevant genes, the patients in the METABRIC cohort were grouped into three different clusters based on immune-cell infiltration. The empirical Bayesian approach within the limma package was applied to determine DEGs among distinct immune phenotypes, and DEGs with adjusted *P* < 0.05 and |log_2_FC| > log_2_1.5 were considered as TME-related genes. The correlation between the relative expression value (Z-Score transformed) of each gene and DSS was evaluated by the univariate Cox regression analysis, and genes with *P* < 0.05 were considered as prognosis-related genes. The genes associated with both TME and prognosis were finally reserved for further analysis.

### Development and Validation of an Immune-Related Gene Signature

The CKI-perturbed genes were intersected with the TME- and prognosis-relevant genes, and subsequently the down-regulated genes with HR > 1 and the up-regulated genes with HR < 1 were reserved. Functional annotation for immune-related genes regulated by CKI was performed by the hypergeometric test. The formula is as follows:


$$P=1-\sum_{i=0}^{m-1}\frac{\left(\frac{M}{i}\right)\left(\frac{N-M}{n-i}\right)}{\left(\frac{N}{n}\right)}$$

where *N* is the number of all genes annotated by GO; *n* is the number of target genes in *N*; *M* is the number of all genes annotated in a specific GO term; *m* is the number of target genes that can be annotated in a specific GO term. After the *P* value was adjusted by the Benjamini-Hochberg correction, GO terms that met adjusted *P* < 0.05 were defined as significantly enriched GO terms. To obtain the most useful immune-related prognostic markers in the training set, the least absolute shrinkage and selection operator (LASSO) penalty within the glmnet package (61) was implemented to reduce dimensionality. Genes represented by an optimal value of the penalty parameter λ (the λ with minimum mean cross-validated error) that was determined by five-fold cross-validation constituted the immune-related gene signature in this study. Finally, these genes were fitted into a multivariate Cox regression model, and the linear combination of the expression value of the gene multiplied by its regression coefficients derived from the multivariate Cox regression model generated a prognostic risk score with the genes. The formula is as follows:


$$Risk\;Score=h_{0}(t)\times e^{\Sigma_{i=1}^{n}\;\beta_{i}x_{i}}$$

where *t* is the survival time, *h_0_* (*t*) is the baseline hazard, *n* is the number of genes, *x_i_* is the expression value of the *i*th gene and *β_i_* is the regression coefficient of the *i*th gene.

The patients were divided into either low- or high-risk group according to the optimal cut-off point determined *via* the maximally selected rank statistics method in the survminer package, and meanwhile the minimal proportion of observations per group was set to 30% to prevent the problem of too few patients in a certain group. The survival curves were generated by the Kaplan-Meier method, and the log-rank test was used to assess the differences between the low- and high-risk groups. Survival predictive accuracy of the prognostic model was estimated through receiver operating characteristic curve (ROC) and Harrell’s concordance index (C-index) analyses. The predictive efficacy of the gene signature for patients’ prognosis was further validated in the test set mentioned above. The univariate and multivariate Cox proportional hazards models were utilized to analyze whether the risk scores had a prognostic significance. Age, tumor size, the number of positive lymph nodes and PAM50 subtypes were firstly evaluated in the univariate Cox proportional hazards model, and all statistically significant variables were then added as covariates in the multivariate Cox proportional hazards model. Further validation on the prognostic value of risk scores was performed by comparing the predictive ability between two models: one comprising tumor size and the number of positive lymph nodes as covariates; and the other including risk scores as the third covariate. The time-dependent C-index and the area under curve (AUC) of time-dependent ROC curve were set as the indicators of prognostic efficacy.

### Statistical Analysis

For two-group comparisons, the Shapiro-Wilk test was applied to assess the assumption of normal distribution, and statistical significance of differences between non-normally distributed variables was estimated with the Wilcoxon test. For comparisons among more than two groups, the Kruskal-Wallis test was utilized as a non-parametric method. Correlation coefficients (*ρ*) were generated by Spearman’s and distance correlation analyses. Survival analysis was performed *via* the Kaplan-Meier method, and differences in survival distributions were evaluated using the log-rank test. The “surv-cutpoint” function in the survminer package, which repeatedly tests all potential cut points to find the maximum rank statistics, was applied to determine the best cut-off level for each prognostic marker. Univariate and multivariate analyses were conducted employing Cox proportional hazard models. All the tests were two sided, and statistical significance was set at *P* < 0.05, unless otherwise stated. The Benjamini-Hochberg correction was applied in multiple tests to reduce false positive rates. All statistical analyses in this study were performed with the R software (version 4.0.3, http://www.R-project.org).

## Results

### Main Constituents of CKI Detected by UPLC-Q-TOF-MS

**Figure 1:**
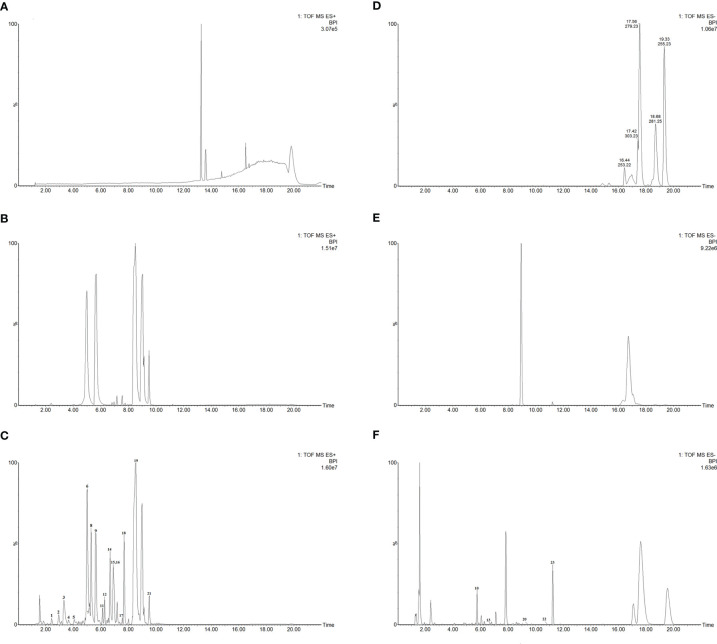
UPLC-Q-TOF-MS analysis of CKI. Chromatographic fingerprints of control **(A)**, standard components **(B)** and CKI **(C)** in the positive ion mode. Chromatographic fingerprints of control **(D)**, standard components **(E)** and CKI **(F)** in the negative ion mode.

In this study, we identified a total of 23 chemical components from CKI by using UPLC-Q-TOF-MS analysis (**Figure 1** and **Table 1**). Consistent with previous findings (37, 62), we also identified the six alkaloids that have been considered as the major active ingredients of CKI, namely, matrine, oxymatrine, N-methylcytisine, sophoridine, sophocarpine and oxysophocarpine.

**Table 1:** Compounds of CKI detected by UPLC-Q-TOF-MS.

Peak No.	Compounds	t_R_ (min)	Formula	Observed mass (Da)	Calculated mass (Da)	Mass error (ppm)	Adducts	MS/MS
**1**	5α,9α-dihydroxymatrine	2.43	C_15_H_25_N_2_O_3_	281.1856	281.1860	-1.4	[M+H]^+^	263.1784, 245.1643, 218.1799, 148.1144, 134.0966
**2**	Oxysophoranol	2.93	C_15_H_25_N_2_O_3_	281.1856	281.1860	-1.4	[M+H]^+^	263.1784, 245.1675, 138.0924
**3**	9α-hydroxymatrine	3.32	C_15_H_25_N_2_O_2_	265.1912	265.1911	0.3	[M+H]^+^	247.1837, 205.1368, 177.1410, 150.1284, 148.1144, 134.0966, 120.0816
**4**	9α-hydroxysophocarpine	3.80	C_15_H_21_O_2_N_2_	261.1607	261.1598	3.4	[M+H]^+^	177.1410, 148.1119
**5**	Cytisine	3.95	C_11_H_15_N_2_O_2_	191.1184	191.1179	2.6	[M+H]^+^	148.0771, 133.0773, 120.0861, 96.0789
**6**	Oxymatrine^1)^	4.99	C_15_H_25_N_2_O_2_	265.1938	265.1911	10.0	[M+H]^+^	247.1837, 205.1368, 176.1104, 148.1144, 136.1134, 120.0839
**7**	Isokuraramine	5.17	C_12_H_18_N_2_O_2_	223.1464	223.1447	7.6	[M+H]^+^	189.1406, 175.1261,
**8**	Oxysophocarpine	5.29	C_15_H_23_N_2_O_2_	263.1759	263.1754	1.8	[M+H]^+^	245.1675, 203.1196, 177.1410, 150.1284, 136.1134
**9**	Sophoridine^1)^	5.63	C_15_H_24_N_2_O	249.1984	249.1961	9.2	[M+H]^+^	247.1837, 218.1496, 190.1240, 180.1411, 150.1284, 148.1144, 112.0781
**10**	Isokuraridin	5.75	C_26_H_30_O_6_	437.1679	437.1669	2.2	[M-H]^-^	301.0666, 287.0548, 244.0340, 243.0312, 149.0443
**11**	14β-hydrsophoridine	6.14	C_15_H_25_O_2_N_2_	265.1905	265.1911	-2.2	[M+H]^+^	164.1083, 148.1119
**12**	Baptifoline	6.28	C_15_H_21_O_2_N_2_	261.1607	261.1598	3.4	[M+H]^+^	243.1485, 115.0962, 114.0930, 96.0829
**13**	Kushenol Q	6.60	C_25_H_30_O_7_	441.1908	441.1918	-2.6	[M-H]^-^	109.0301
**14**	N-methylcytisine	6.67	C_12_H_17_N_2_O	205.1338	205.1335	1.4	[M+H]^+^	146.0621, 108.0830
**15**	Sophoranol	6.80	C_15_H_25_O_2_N_2_	265.1938	265.1911	10.0	[M+H]^+^	247.1805, 150.1309, 148.1144,
**16**	Lupanine	6.90	C_15_H_25_N_2_O	249.1984	249.1961	9.2	[M+H]^+^	231.1861, 166.1249, 150.1309, 148.1144, 136.1134, 122.0991, 114.0930
**17**	Oxysophoridine	7.56	C_15_H_25_N_2_O_2_	265.1938	265.1911	10.0	[M+H]^+^	247.1837, 245.1658, 188.1485, 168.1395, 150.1281, 148.1117, 122.0954, 112.0772
**18**	Lamprolobine	7.69	C_15_H_25_N_2_O_2_	265.1912	265.1911	-0.3	[M+H]^+^	247.1837, 245.1675, 188.1457, 150.1284, 148.1144,122.0991
**19**	Matrine^1)^	8.49	C_15_H_24_N_2_O	249.1984	249.1961	9.2	[M+H]^+^	247.1837, 176.1077, 150.1284, 148.1144
**20**	Formononetin	9.40	C_16_H_12_O_4_	267.0659	267.0650	3.3	[M-H]^-^	267.0626, 252.0430, 223.0327
**21**	Sophocarpine^1)^	9.50	C_15_H_22_N_2_O	247.1805	247.1805	0	[M+H]^+^	245.1675, 179.1566, 150.1284, 148.1144, 136.1134
**22**	8-lavandulyl kaempferol	10.79	C_26_H_30_O_5_	421.2064	421.2020	10.0	[M-H]^-^	379.1642, 271.0573, 149.0493
**23**	Maackiain	11.25	C_16_H_12_O_5_	283.0606	283.0600	2.1	[M-H]^-^	255.0638, 240.0447, 211.0762, 185.0567, 137.0223

^1)^ standard components.

### Data Analysis for the Drug-Perturbed Cell Line Samples

**Figure 2:**
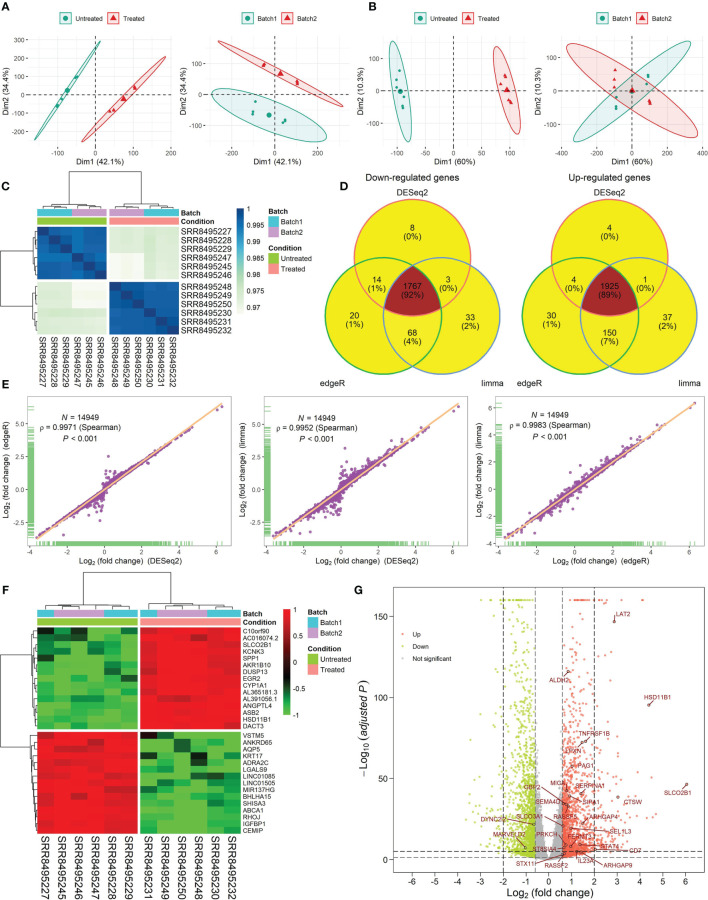
RNA-seq data analysis for six CKI-perturbed and six control cell line samples. **(A, B)** PCA for the transcriptome profiles before removing batch effects **(A)** and after removing batch effects **(B)**. **(C)** Hierarchical clustering of 12 samples using Spearman distance. **(D)** Venn diagram of DEGs calculated by three methods. **(E)** High correlation of log_2_FC calculated by three methods. The correlation coefficient (*ρ*) is generated by Spearman’s correlation. **(F)** Hierarchical clustering of consistent DEGs detected by three methods. Top 15 up- and down-regulated DEGs are shown in the heatmap. **(G)** Volcano plot of DEGs. Log_2_FC and *P* values calculated by DESeq2 were used. The red dot represents up-regulated genes (adjusted *P* < 0.05 and log_2_FC > log_2_1.5) and the green dot represents down-regulated genes (adjusted *P* < 0.05 and log_2_FC < -log_2_1.5). A total of 28 TME- and prognosis-relevant genes perturbed by CKI were labeled.

The results of RNA-seq data analysis for the CKI-perturbed cell line samples were shown in **Figure 2** and **Supplementary Tables 4–6**. The PCA showed the difference in the sample distribution before or after removing batch effects (**Figures 2A, B**). The hierarchical clustering presented a strong intra-group correlation and a relatively low inter-group correlation (**Figure 2C**). We found 1925 consistent up-regulated genes and 1767 consistent down-regulated genes between CKI-treated and control groups (**Figure 2D**), and Pearson’s correlation analysis exhibited high correlation of log_2_FC calculated by three methods. The heatmap and volcano plots for the DEGs were displayed in **Figures 2F, G**.

### Pathways Regulated by CKI

**Figure 3:**
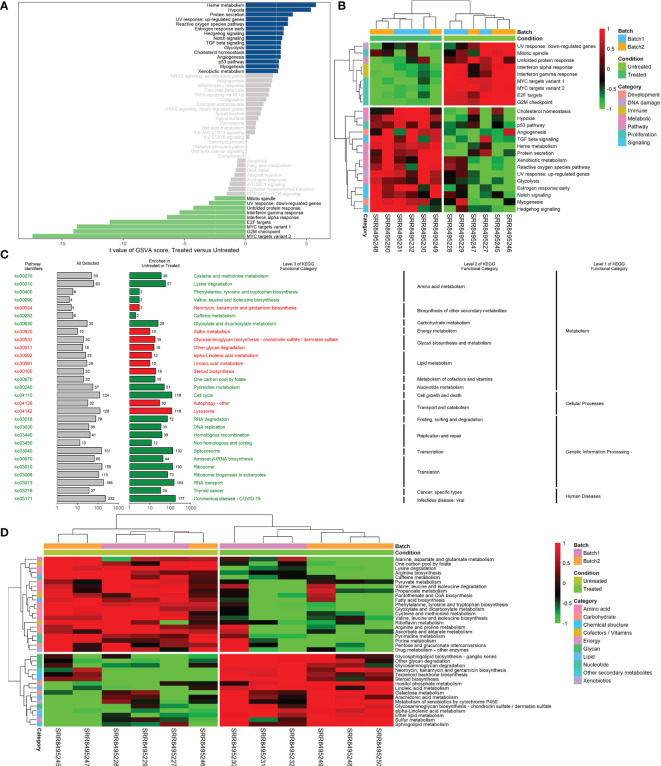
Differences in hallmark or KEGG pathway activities scored per sample by GSVA between CKI-treated and control groups. **(A)** Differential hallmark pathway activities in CKI-treated versus control groups. **(B)** Clustering results of hallmark pathway activities between CKI-treated and control groups. The heatmap visualizes differential hallmark gene sets (adjusted *P* < 0.05). **(C)** Significantly different KEGG functional categories and pathways between CKI-treated and control groups. The pathways with |log_2_FC| > 0.5 and adjusted *P* < 0.05 are visualized. KEGG functional categories and pathways that were significantly enriched in the CKI-treated group are shown in red; those significantly enriched in the control group are shown in green. **(D)** Clustering results of KEGG metabolic pathway activities between CKI-treated and control groups. The heatmap visualizes differential KEGG metabolic pathway (adjusted *P* < 0.05).

**Figure 4:**
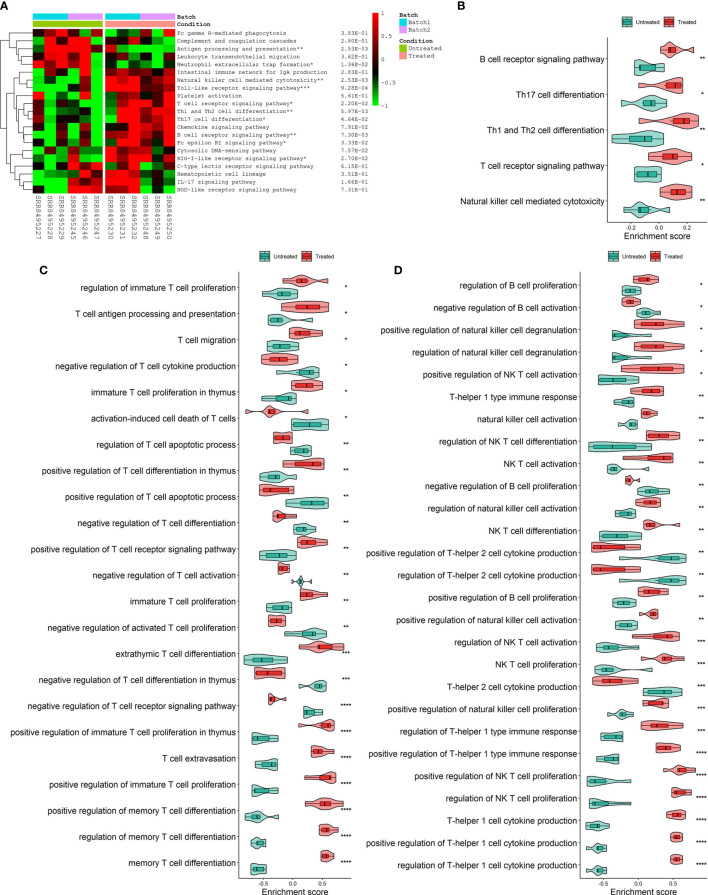
Differences in immune-related process activities scored per sample by GSVA between CKI-treated and control groups. **(A)** Clustering results of KEGG immune system pathway activities between CKI-treated and control groups. **(B)** Main differential KEGG immune system pathways activated in the CKI-treated group (*P* < 0.05). **(C)** Significantly different GO biological processes (adjusted *P* < 0.05) correlated with T cells between CKI-treated and control groups. **(D)** Significantly different GO biological processes (adjusted *P* < 0.05) correlated with Th1, Th2, B, NK and NK T cells between CKI-treated and control groups (*0.01 < *P* < 0.05; **0.001 < *P* < 0.01; ***0.0001 < *P* < 0.001; ****P < 0.0001).

Analysis of hallmark pathway gene signatures highlighted that cell cycle-related pathways like MYC targets, G2M checkpoint and E2F targets were significantly down-regulated in the CKI treatment group compared with the control group (**Figures 3A, B**). According to the GSVA results of KEGG pathways (**Figure 3C**), 113 biological pathways (including 53 activated and 60 suppressed) were significantly changed between CKI-treated and control groups, which exhibited a comprehensive influence of CKI on metabolism, cellular processes, genetic and environmental information processing, and organismal systems. Metabolic pathway analysis showed CKI decreased pathways involved in nucleotide metabolism (purine and pyrimidine metabolism) and lipid metabolism (fatty acid biosynthesis) (**Figure 3D**). Especially, the KEGG pathways associated with immune system such as natural killer cell mediated cytotoxicity, T cell receptor signaling pathway, Th1 and Th2 cell differentiation, Th17 cell differentiation and B cell receptor signaling pathway were activated in the CKI-treated group versus the control group, which suggested its potential effects on immunoregulation (**Figures 4A, B**). Analysis of GO gene signatures highlighted that most of biological processes that promote immune response were activated after CKI treatment (**Figures 4C, D**). Compared with the samples in the control group, most biological processes that improve T cell proliferation, differentiation and T cell receptor signaling pathway or induced cell death of T cells were activated, whereas those that inhibit T cell activation, proliferation, differentiation and T cell receptor signaling pathway were inactivated in the CKI-perturbed samples (**Figure 4C**). Meanwhile, the activation and proliferation of NK, NKT and B cells were up-regulated after CKI treatment. Notably, for the Th1 cells, which execute anti-tumor immunity functions, multiple processes that promote their activities such as positive regulation of T-helper 1 cell cytokine production and positive regulation of T-helper 1 type immune response were positively regulated in the CKI-treated group. However, for the Th2 cells, which executing pro-tumor, immune suppressive functions, the cell cytokine production of Th2 cells was down-regulated in the CKI-treated group (**Figure 4D**).

### Immune Subtypes and Immune-Related Genes

**Figure 5:**
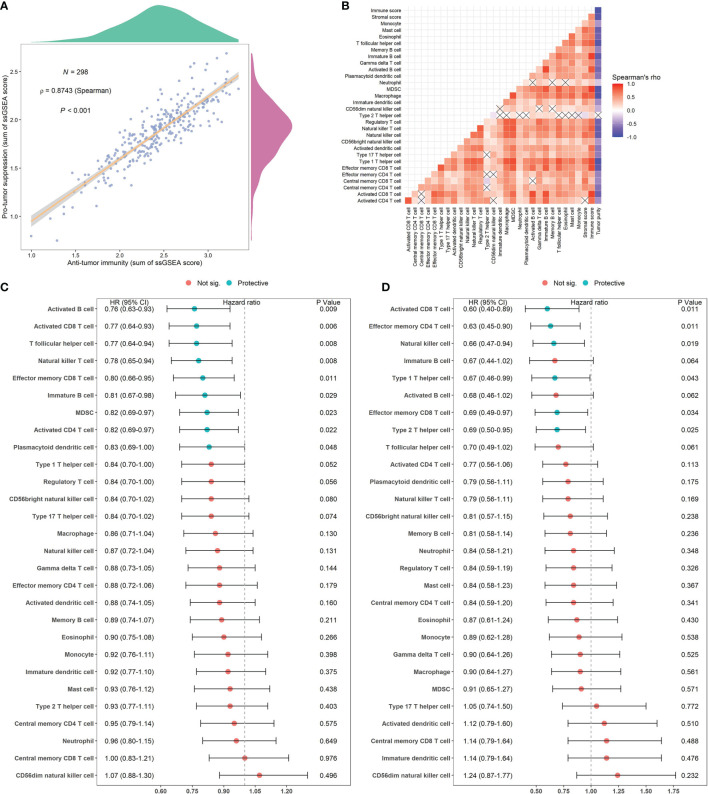
Correlations of microenvironment cells and prognostic significance of them in TNBC. **(A)** Correlations between infiltration of cell types executing anti-tumor immunity (activated CD4^+^ T cells, activated CD8^+^ T cells, CD4^+^ Tcm cells, CD8^+^ Tcm cells, CD4^+^ Tem cells, CD8^+^ Tem cells, Th1 cells, Th17 cells, activated DCs, CD56bright NK cells, NK cells, NKT cells) and cell types executing pro-tumor, immune suppressive functions (Treg, Th2 cells, CD56dim NK cells, immature DCs, macrophages, MDSCs, neutrophils, and pDCs). The correlation coefficient (*ρ*) is generated by Spearman’s correlation. The shaded area represents 95% confident interval. **(B)** Correlations of the relative abundance of tumor-infiltrating immune cells using Spearman analysis. The stromal score, immune score and tumor purity were also plotted. A negative correlation is marked with blue and a positive correlation with red. “×” means the corresponding correlation coefficient is regarded as insignificant (*P* > 0.05). **(C, D)** Estimation of the prognostic value of each cell subset by using a univariate Cox proportional hazards model for the METABRIC cohort **(C)** and the GSE58812 cohort **(D)**.

**Figure 6:**
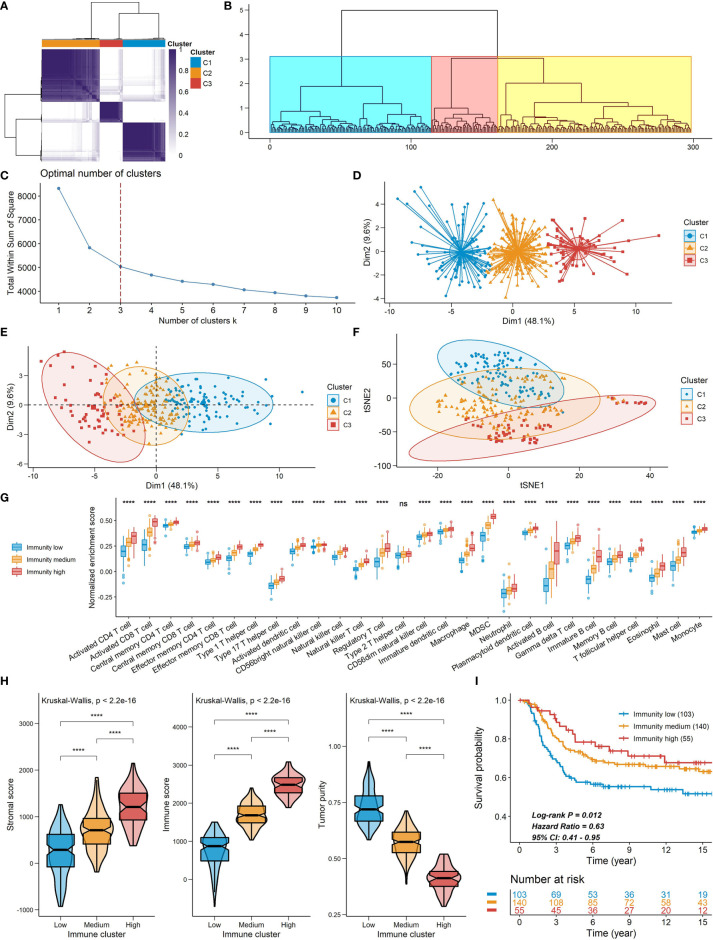
Constructions of TME signatures in the METABRIC cohort. **(A, B, D)** Consensus clustering **(A)**, hierarchical clustering **(B)** and k-means clustering **(D)** of TNBC patients based on tumor-infiltrating immune cells to classify patients into three groups. **(C)** Screening for the optional number of clusters with k-means clustering. **(E, F)** PCA **(E)** and t-SNE **(F)** for dimensional reduction of TNBC patients based on tumor-infiltrating immune cells. The three clusters detected by the consensus clustering were shown. **(G)** The relative abundance of tumor-infiltrating immune cells in the three immune clusters. **(H)** A comparison of stromal score, immune score and tumor purity among the three clusters. **(I)** Kaplan-Meier curves of the three immune clusters. Plots were truncated at 15 years, but the analyses were conducted using all of the data ****P < 0.0001; ns, *P* > 0.05).

**Figure 7:**
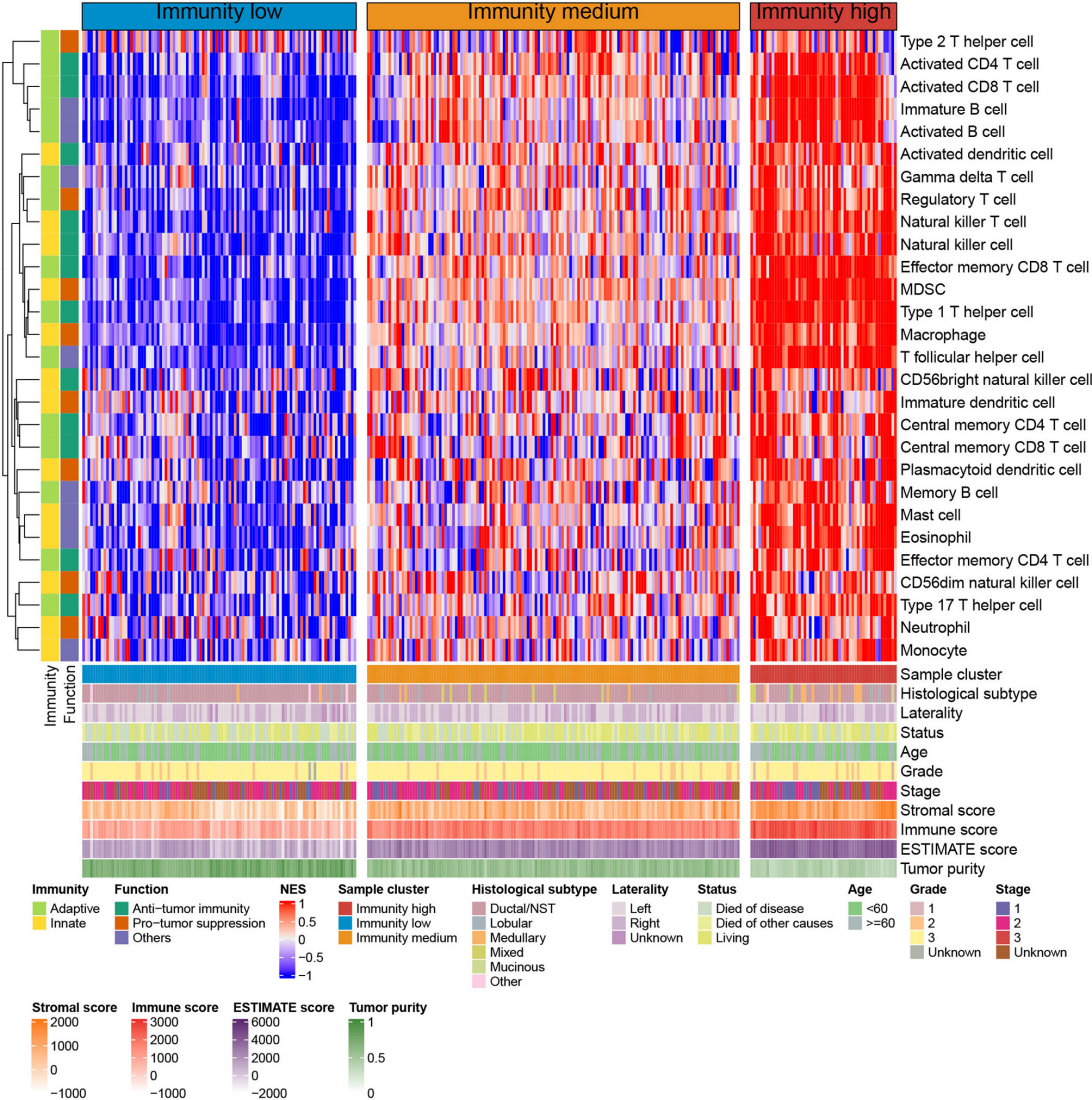
Unsupervised clustering of TNBC microenvironment phenotypes in the METABRIC cohort. Rows represent tumor-infiltrating immune cells and columns represent samples.

**Figure 8:**
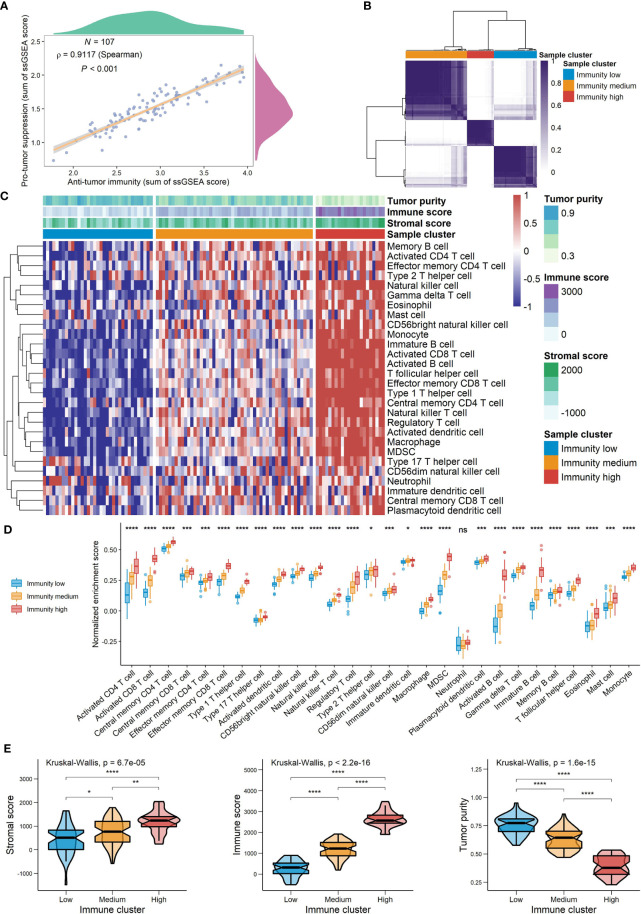
The landscape of TME signatures in the GSE58812 cohort. **(A)** Correlations between infiltration of cell types executing anti-tumor immunity and cell types executing pro-tumor, immune suppressive functions. The correlation coefficient (*ρ*) is generated by Spearman’s correlation. The shaded area represents 95% confident interval. **(B)** Consensus clustering of TNBC patients based on tumor-infiltrating immune cells to classify patients into three groups. **(C)** Unsupervised clustering of TNBC microenvironment phenotypes. Rows represent tumor-infiltrating immune cells and columns represent samples. **(D)** The relative abundance of tumor-infiltrating immune cells in the three immune clusters. **(E)** A comparison of stromal score, immune score and tumor purity among the three clusters (*0.01 < *P* < 0.05; **0.001 < *P* < 0.01; ***0.0001 < *P* < 0.001; ****P < 0.0001; ns, *P* > 0.05).

**Figure 9:**
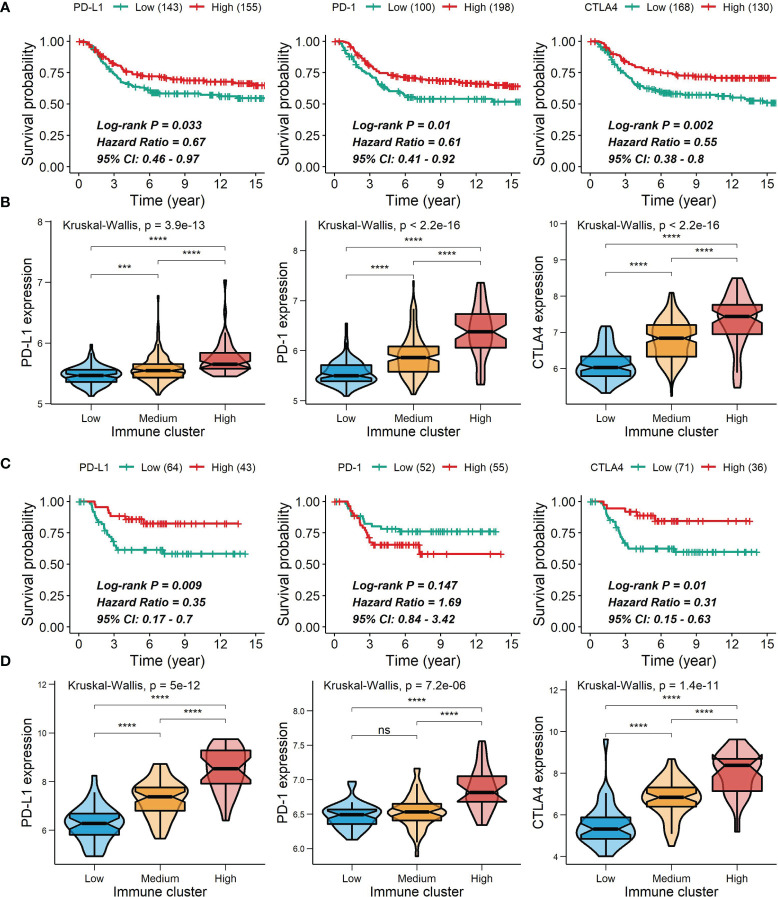
Correlations of PD-L1, PD-1 and CTLA4 expression with survival and comparison of PD-L1, PD-1 and CTLA4 expression among the immune clusters. **(A, C)** Correlations of PD-L1, PD-1 and CTLA4 expression with survival in the METABRIC cohort **(A)** or the GSE58812 cohort **(C)**. **(B, D)** Differences in PD-L1, PD-1 and CTLA4 expression among the immune clusters in the METABRIC cohort **(B)** or the GSE58812 cohort **(D)** ***0.0001 < *P* < 0.001; ****P < 0.0001; ns, *P* > 0.05).

Spearman’s correlation analysis showed that the abundance of cells executing anti-tumor reactivity was positively associated with the abundance of cells delivering pro-tumor suppression (**Figures 5A**, **8A**). The universal landscape of immune cell interaction in TME was visualized in **Figure 5B**, which demonstrated that the relative abundance of most infiltrating immune cell populations was positively correlated with each other, stromal scores and immune scores and negatively correlated with tumor purity. Prognostic significance of each microenvironment cell in TNBC was shown in **Figures 5C, D**. Based on the enrichment scores of 28 gene signatures for the 298 TNBC samples, we performed unsupervised clustering to classify these patients into three independent subtypes (we named them as immunity low, immunity medium and immunity high) (**Figures 6A–F**, **8B**). The three clusters witnessed a remarkable difference in the relative infiltration of immune cell populations (**Figures 7**, **6G**, **8C, D**), stromal scores, immune scores and tumor purity (**Figures 6H**, **8E**). Considering the vital role of TME in prognosis, we investigated the clinical relevance of the immune clusters. Kaplan-Meier analysis exhibited significant associations between immune clusters and DSS (log-rank test, *P* = 0.012), and the immunity high or medium cluster had a longer survival time than the immunity low cluster (**Figure 6I**). The multivariate Cox proportional hazards model also disclosed that the immune clusters independently predicted a better DSS in TNBC (Immunity medium: HR, 0.55; 95% confidence interval, 0.36-0.82; *P* = 0.004; Immunity high: HR, 0.47; 95% confidence interval, 0.26-0.84; *P* = 0.011; **Table 2**). We also analyzed the three crucial immune checkpoints PD-L1, PD1 and CTLA4 in each immunity subtype. The immunity high cluster was featured by a significantly higher PD-L1/PD1/CTLA4 expression levels while the immunity low cluster with a lower PD-L1/PD1/CTLA4 expression levels (**Figure 9**). After conducting differential expression analysis, we found 1591 DEGs between the immunity high and immunity low groups, and 313 between the immunity medium and immunity low groups (**Supplementary Table 7**). We aggregated these genes and finally identified 1593 unique genes as immune-related genes in TNBC.

**Table 2:** HRs and *P* values of the covariates in the univariate and multivariate Cox proportional hazards model for DSS.

Variables		Univariate		Multivariate	
		HR (95% CI)	*P*	HR (95% CI)	*P*
**Age**		1 (0.98-1.01)	0.638	Not included	
**Tumor size**		1.01 (1-1.02)	0.005	1 (1-1.01)	0.221
**Positive lymph nodes**		1.13 (1.09-1.16)	< 0.001	1.13 (1.1-1.17)	< 0.001
**PAM50 subtypes**	**Basal**	Ref		Not included	
	**Nonbasal**	0.79 (0.54-1.14)	0.208		
**Immune clusters**	**Immunity low**	Ref		Ref	
	**Immunity medium**	0.62 (0.42-0.93)	0.021	0.55 (0.36-0.82)	**0.004**
	**Immunity high**	0.5 (0.28-0.89)	0.019	0.47 (0.26-0.84)	**0.011**

### Key CKI-Perturbed Immune-Related Genes and Their Prognostic Values

**Figure 10:**
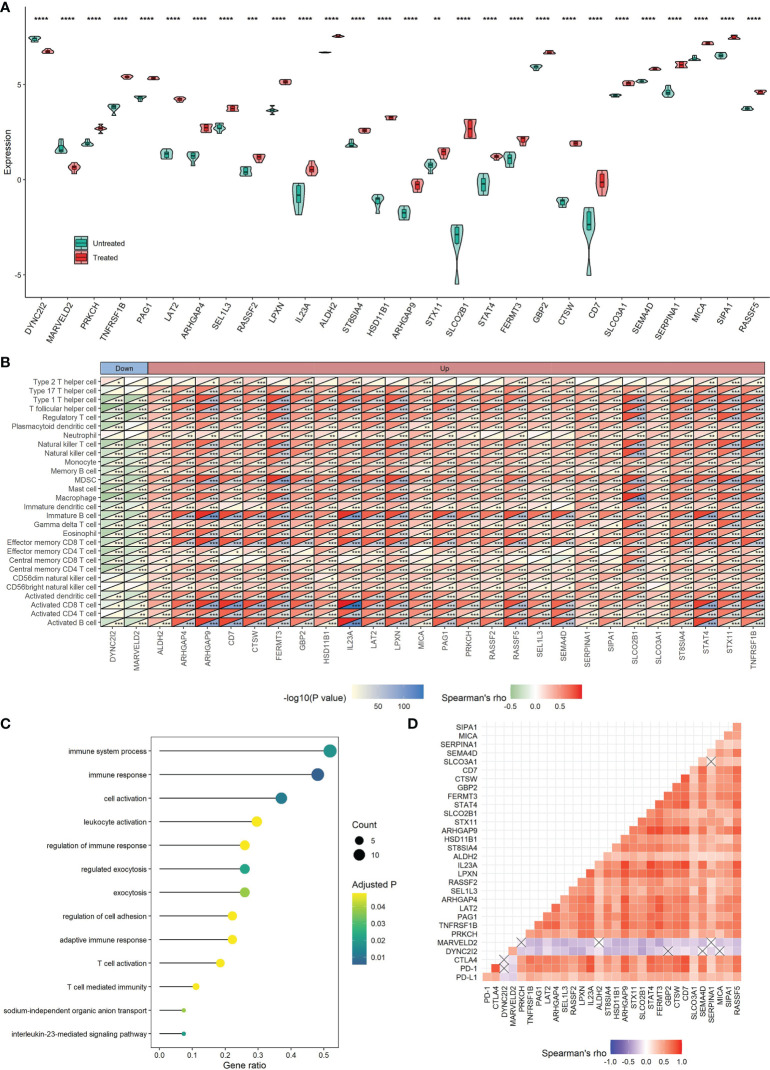
Roles of 28 TME-relevant genes perturbed by CKI. **(A)** Differential expression of 28 TME-relevant genes between CKI-treated and control groups. The *P* values were generated by the DESeq2 method. **(B)** Associations between the expression of 28 TME-relevant genes and the relative abundance of 28 tumor-infiltrating immune cells using Spearman’s correlation based on the METABRIC cohort (*0.01 < *P* < 0.05; **0.001 < *P* < 0.01; ***0.0001 < *P* < 0.001; ****P < 0.0001; ns, *P* > 0.05). **(C)** GO enrichment analysis for 28 TME-relevant genes. A total of 13 significant biological processes with adjusted *P* < 0.05 are shown. **(D)** Correlations of the expression of 28 TME-related genes using Spearman analysis based on the METABRIC cohort. PD-L1, PD-1 and CTLA4 were also plotted. Negative correlation is marked with blue and positive correlation with red. “×” means the corresponding correlation coefficient is regarded as insignificant (*P* > 0.05).

For the 1593 immune-related genes in TNBC, univariate Cox analysis showed 304 of them were correlated with DSS time (*P* < 0.05), in which two genes (DYNC2I2 and MARVELD2) with HR > 1 can be down-regulated by CKI and 26 genes (PRKCH, TNFRSF1B, PAG1, LAT2, ARHGAP4, SEL1L3, RASSF2, LPXN, IL23A, ALDH2, ST8SIA4, HSD11B1, ARHGAP9, STX11, SLCO2B1, STAT4, FERMT3, GBP2, CTSW, CD7, SLCO3A1, SEMA4D, SERPINA1, MICA, SIPA1 and RASSF5) with HR < 1 can be up-regulated by CKI (**Figure 10A**). In the present study, these 28 genes were considered as key TME- and prognosis-related genes regulated by CKI. Clearly, the 26 genes were positively associated with the relative abundance of most infiltrating immune cell populations, while the other two genes showed negative associations (**Figure 10B**). GO functional annotation presented that the 28 CKI-perturbed immune-related genes were significantly enriched in immune system process, immune response, regulation of immune response, adaptive immune response, T cell activation and T cell mediated immunity (**Figure 10C**). The expression of the 26 genes that up-regulated by CKI were positively correlated with each other, PD-L1, PD-1 and CTLA4, while the expression of MARVELD2 and WDR34 that down-regulated by CKI were negatively correlated with the other 26 genes (**Figure 10D**). These genes were enrolled to construct a prognostic signature.

**Figure 11:**
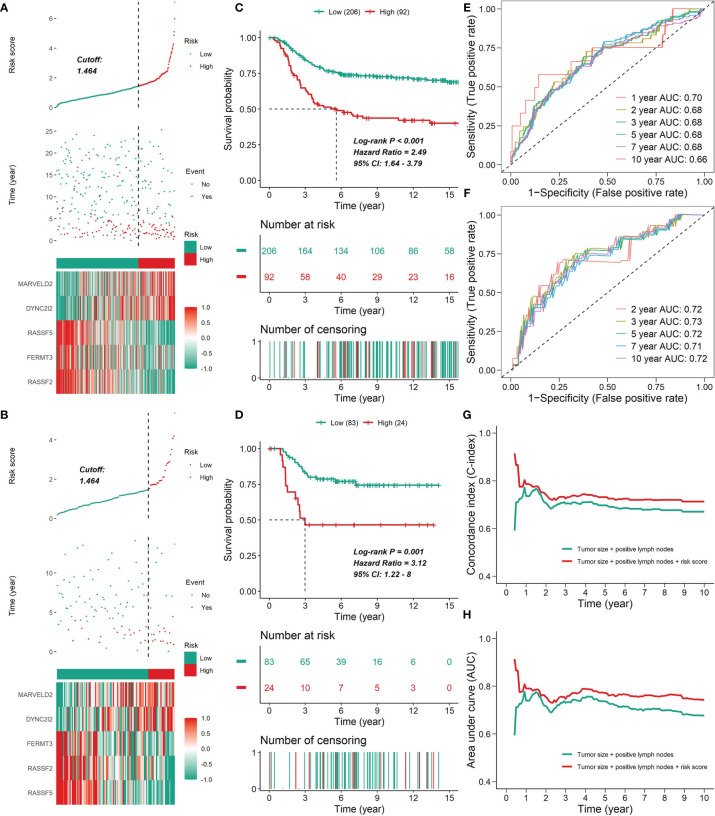
The prognostic value of the five-gene signature. **(A, B)** The distribution of risk scores, survival time, status and prognostic gene expression patterns for the 298 patients in the testing set **(A)** or the 107 patients in the test set **(B)**. For the METABRIC cohort, plots were truncated at 15 years, but the analyses were conducted using all of the data. **(C, D)** Kaplan-Meier curves of patients in the high- and low-risk groups in the METABRIC cohort **(C)** or the GSE58812 cohort **(D)**. **(E, F)** ROC analysis of the five-gene signature for prediction of survival risk in the training set **(E)** or the test set **(F)**. **(G, H)** Time-dependent C-index curves **(G)** or time-dependent ROC curves **(H)** with two Cox proportional hazards models for DSS (based on the METABRIC cohort); one included two covariates (tumor size and number of positive lymph nodes), and the other added immune clusters as covariates. The C-index was internally validated using Bootstrap cross validation for 1000 times. The significant difference in the C-index and AUC was observed after one year.

**Figure 12:**
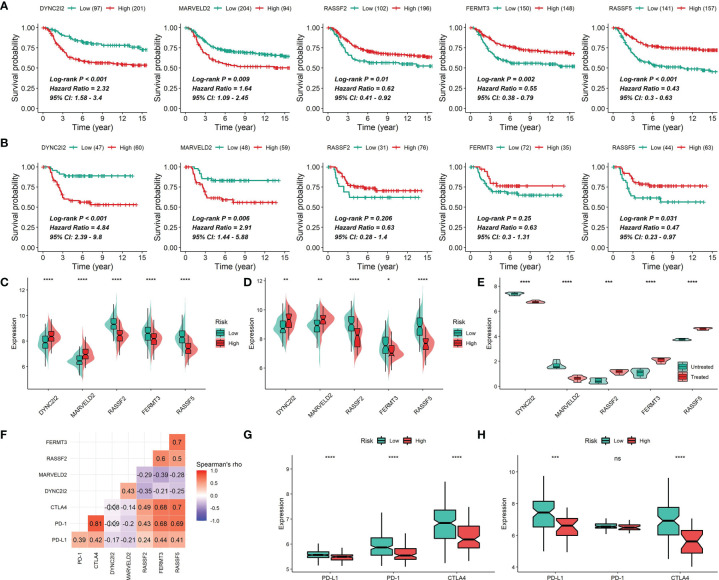
Gene expression of DYNC2I2, MARVELD2, RASSF2, FERMT3 and RASSF5. **(A, B)** Correlations of DYNC2I2, MARVELD2, RASSF2, FERMT3 and RASSF5 expression with survival time in the METABRIC cohort **(A)** or the GSE58812 cohort **(B)**. **(C, D)** Correlations of DYNC2I2, MARVELD2, RASSF2, FERMT3 and RASSF5 expression with survival risks in the METABRIC cohort **(C)** or the GSE58812 cohort **(D)**. **(E)** Differential expression of DYNC2I2, MARVELD2, RASSF2, FERMT3 and RASSF5 between CKI-treated and control groups. The *P* values were generated by the DESeq2 method. **(F)** Correlations of the expression of DYNC2I2, MARVELD2, RASSF2, FERMT3 and RASSF5 using Spearman analysis based on the METABRIC cohort. PD-L1, PD-1 and CTLA4 were also plotted. Negative correlation is marked with blue and positive correlation with red. “×” means the corresponding correlation coefficient is regarded as insignificant (*P* > 0.05). (G and H) Difference in PD-L1, PD-1 and CTLA4 expression among risk groups in the METABRIC cohort **(G)** or the GSE58812 cohort **(H)** (*0.01 < *P* < 0.05; **0.001 < *P* < 0.01; ***0.0001 < *P* < 0.001; ****P < 0.0001; ns, *P* > 0.05).

**Figure 13:**
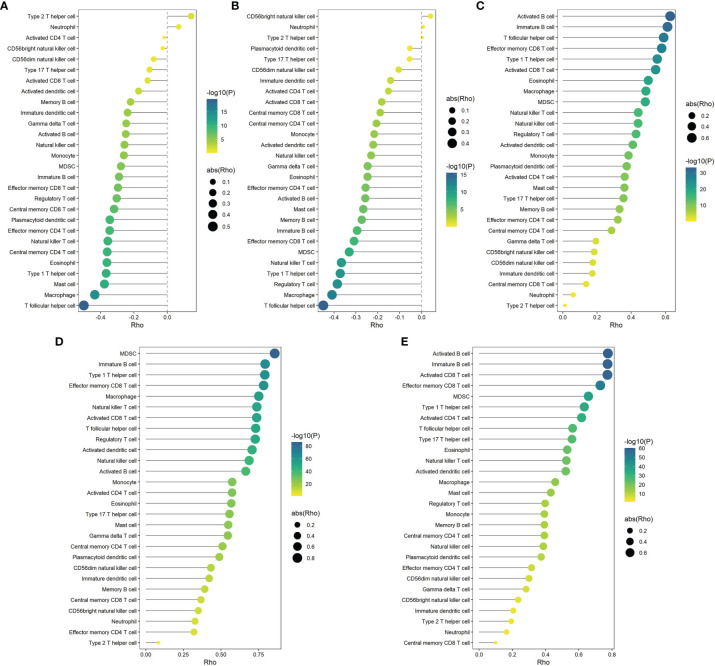
Correlations of the expression of DYNC2I2 **(A)**, MARVELD2 **(B)**, RASSF2 **(C)**, FERMT3 **(D)** and RASSF5 **(E)** with 28 microenvironment cells (based on the METABRIC cohort). Correlation coefficients were calculated by Spearman analysis. The color represents *P* values, and the size of the circles represents the absolute value of correlation coefficients. Larger circles represent bigger correlations.

After performing LASSO and multivariate Cox regression analysis, a five-gene signature correlated with prognosis was constructed, in which two genes (DYNC2I2 and MARVELD2) with HR > 1 were considered as risky genes and three genes (RASSF2, FERMT3 and RASSF5) with HR < 1 were considered as protective genes (**Figures 11**–**13**). The regression coefficient for each gene was also generated, and the survival risk score was computed as follows: risk score = (0.1202 × expression level of DYNC2I2) + (0.2372 × expression level of MARVELD2) + (-0.3560 × expression level of RASSF2) + (0.4986 × expression level of FERMT3) + (-0.5813 × expression level of RASSF5). For the training set, the 206 patients with risk scores higher than the cutoff value (1.464) were classified into the high-risk group, while the rest 92 patients were classified into the low-risk group (**Figure 11A**). The Kaplan-Meier survival analysis exhibited that patients in the high-risk group had shorter survival time in comparison with patients in the low-risk group (Log-rank test *P* < 0.001), suggesting the expression levels of the five genes could effectively discriminate the survival risks of these TNBC patients (**Figure 11C**). The C-index was 0.646 in the training set, and the AUC of the ROC curve was 0.70, 0.68, 0.68, 0.68, 0.68 and 0.66 for 1-year, 2-year, 3-year, 5-year, 7-year and 10-year DSS, respectively, confirming a good predictive efficacy of the prognostic gene signature (**Figure 11E**). For the test set, the 83 patients with risk scores higher than the cutoff value (1.464) were classified into the high-risk group, while the rest 24 patients were included into the low-risk group (**Figure 11B**). The Kaplan-Meier survival analysis showed that patients in the high-risk group had shorter survival time in comparison with patients in the low-risk group (Log-rank test *P* = 0.001), suggesting the expression levels of the five genes could effectively discriminate the survival risks of these TNBC patients (**Figure 11D**). The C-index was 0.696 in the test set, and the AUC of the ROC curve was 0.72, 0.73, 0.72, 0.71 and 0.72 for 2-year, 3-year, 5-year, 7-year and 10-year MFS, respectively, confirming a good predictive efficacy of the prognostic gene signature (**Figure 11F**). The multivariate Cox proportional hazards model also revealed that the risk score independently predicted a worse DSS in TNBC (HR, 1.51; 95% confidence interval, 1.27-1.81; *P* < 0.001; **Table 3**). The time-dependent C-index and the time-dependent ROC curve demonstrated that the addition of risk scores into the Cox proportional hazards model enhanced the prognostic efficacy (**Figures 11G, H**). Higher expression of DYNC2I2 and MARVELD2 was correlated with worse prognosis, whereas higer expression of RASSF2, FERMT3 and RASSF5 was correlated with better prognosis (**Figures 12A, B**). As shown in **Figures 12C, D**, DYNC2I2 and MARVELD2 were up-regulated in the high-risk group versus the low-risk group, and RASSF2, FERMT3 and RASSF5 were down-regulated in the high-risk group versus the low-risk group. The expression of RASSF2, FERMT3 and RASSF5 that up-regulated by CKI were positively correlated with each other, PD-L1, PD-1 and CTLA4, whereas the expression of MARVELD2 and WDR34 that down-regulated by CKI were negatively correlated with RASSF2, FERMT3 and RASSF5 (**Figures 12E, F**). We also analyzed the expression of PD-L1, PD-1 and CTLA4 in the high- and low-risk groups. The low-risk group was characterized by a significantly higher PD-L1/PD1/CTLA4 expression levels, while the high-risk group with a lower PD-L1/PD1/CTLA4 expression levels (**Figures 12G, H**).

**Table 3:** HRs and *P* values of the covariates in the univariate and multivariate Cox proportional hazards model for the risk score.

Variables		Univariate		Multivariate	
		HR (95% CI)	*P*	HR (95% CI)	*P*
**Age**		1 (0.98-1.01)	0.638	Not included	
**Tumor size**		1.01 (1-1.02)	0.005	1 (0.99-1.01)	0.733
**Positive lymph nodes**		1.13 (1.09-1.16)	<0.001	1.12 (1.09-1.16)	<0.001
**PAM50 subtypes**	**Basal**	Ref		Not included	
	**Nonbasal**	0.79 (0.54-1.14)	0.208		
**Risk score**		1.51 (1.28-1.78)	<0.001	1.51 (1.27-1.81)	**<0.001**

## Discussion

CKI has been approved by NMPA to treat cancer-induced pain (11), and the extensive use alone or in combination with chemotherapy or radiotherapy of CKI in the treatment of breast cancer has witnessed remarkable therapeutic and prognostic benefits (12, 16). Meanwhile, a recent study has proved that CKI reshapes the immune microenvironment of hepatocellular carcinoma (HCC) by regulating macrophages and CD8^+^ T cells (10). Multiple studies have revealed the mechanism of CKI on TNBC. CKI strongly reduces the migration and invasion of MDA-MB-231 cells and induces cell cycle arrest and apoptosis in them (36, 37, 63, 64). Furthermore, CKI has been found to suppress energy metabolism and DNA repair pathways in MDA-MB-231 cells (63). However, the effects of CKI on TNBC microenvironment are not fully understood.

In this study, we firstly analyzed the RNA-seq data of CKI-perturbed TNBC cells and detected 3692 differential genes, and later found CKI significantly regulated biological pathways correlated with cell cycle, metabolism and immunity using the GSVA algorithm. We then estimated the relative quantitative infiltration levels of 28 immune cell signatures in TNBC patients from the METABRIC cohort by the ssGSEA algorithm based on gene expression data. Meanwhile, we classified these patients into three distinct immune cell infiltration patterns with the consensus clustering method and then detected 1593 DEGs among these subgroups. Furthermore, we performed univariate Cox analysis and selected two genes with HR > 1 that can be down-regulated by CKI and 26 genes with HR < 1 that can be up-regulated by CKI. Finally, we constructed a prognostic signature of five immune-related genes with the LASSO regression method to predict mortality risks in TNBC patients, and we also confirmed the predictive capability of this immune gene signature in another TNBC cohort. Together, this study detected a signature comprising five immune-related genes regulated by CKI, which could predict the outcomes of TNBC patients with a good performance, and these genes have potential to serve as immune-related biomarkers of CKI on TNBC.

Analysis of hallmark pathway gene signatures found that MYC targets served as the top enriched pathway and it can be remarkably inactivated in the CKI treatment group. Earlier studies indicated that c-Myc is essential for tumor angiogenesis (65). Furthermore, metabolic pathway analysis presented that notably up-regulated pathways such as purine metabolism, pyrimidine metabolism and fatty acid biosynthesis in TNBC patients (66) were inhibited after CKI treatment. Eventually, analysis of immunity-related signatures highlighted that CKI could up-regulate pathways enhancing T, B, NK and NKT cell activities and down-regulate pathways inhibiting these cells’ functions, which suggested the immunomodulatory potential of CKI.

We found two genes (MARVELD2 and WDR34) with HR > 1 can be down-regulated by CKI and 26 genes (GBP2, LPXN, ALDH2, SLCO2B1, SIPA1, SERPINA1, RASSF2, PRKCH, SEMA4D, FERMT3, TCRVB, RASSF5, ARHGAP9, ARHGAP4, SEL1L3, LAT2, SLCO3A1, TNFRSF1B, STAT4, MICA, STX11, PAG1, CD7, ST8SIA4, HSD11B1 and CTSW) with HR < 1 can be up-regulated by CKI. Meanwhile, these genes were involved in multiple biological processes relevant to immunoregulation, such as immune system process, immune response, regulation of immune response, adaptive immune response, T cell activation and T cell mediated immunity. Therefore, we considered that CKI might increase immune activities and improve patients’ prognosis by down-regulating the risky genes and up-regulating the protective genes. Interestingly, all the 26 genes that up-regulated by CKI were positively correlated with PD-L1, PD-1 and CTLA4, which suggested that CKI might have potential to enhance the patients’ sensitivity for immune checkpoint inhibitors when combined with them.

The five-gene signature that we built for predicting patient outcome consisted of two risky genes (MARVELD2 and DYNC2I2) and three protective genes (RASSF2, FERMT3 and RASSF5). For the two risky genes, the prognostic value of DYNC2I2 in breast cancer has been studied in former works, while that of MARVELD2 has not. For DYNC2I2 (also known as WDR34), an integrated bioinformatics study shows that high DYNC2I2 mRNA expression is correlated with shorter overall survival and relapse-free survival in breast cancer patients (67), which is consistent with our finding that DYNC2I2 could serve as a risky factor in TNBC. DYNC2I2 is also found to play oncogenic roles in the progression of HCC (68), whereas it is shown as a tumor-suppressor molecule in human oral cancer (69). With regard to MARVELD2, pathogenic mutations of this gene cause autosomal recessive non-syndromic hearing loss (DFNB49) (70), nevertheless, little is known about the roles of MARVELD2 in cancer.

As for the three protective genes, the prognostic value of FERMT3 and RASSF2 has been noted in breast cancer, while that of RASSF5 has not. With regard to FERMT3 (also known as Kindlin-3), whether this gene plays a suppressing or promoting role in breast cancer progression is still controversial. It has been reported that reduced expression of FERMT3 in breast cancer promotes metastasis formation by mediating β3-integrin activation (71). Conversely, this gene is also found to enhance breast cancer progression and metastasis by activating Twist-mediated angiogenesis (72). Moreover, FERMT3 expressed in the TME is correlated with a poor prognosis of breast cancer patients (73), which is inconsistent with our finding that FERMT3 could serve as a protective prognostic factor in TNBC. These results highlight the complex role of FERMT3 which could exhibit dual effects, so the prognostic value of this gene in breast cancer deserve more attention. RASSF2 and RASSF5 belong to the RASSF family that shares a region of homology (the Ras association domain), and RASSF proteins functions as tumor suppressors by interacting either directly or indirectly with activated Ras and regulating cell growth signaling (74). RASSF2 has been reported as a tumor suppressor to be frequently inactivated by promoter methylation in breast cancer and to inhibit the growth of breast cancer cell lines both *in vitro* and *in vivo* (75). Contrarily, although RASSF2 hypermethylation predicts a worse prognosis in squamous cervical cancer (76), nasopharyngeal carcinoma, gastric cancer and Ewing sarcoma, it has also been shown as an independent indicator of better prognosis (77) in breast cancer, which is inconsistent with our finding that high RASSF2 levels might be a protective prognostic factor in TNBC. Therefore, more studies in larger cohorts on the clinical involvement of RASSF2 in breast cancer should be performed. With regard to RASSF5 (also called NORE1), this gene encodes at least three distinct isoforms and is clearly regarded as a Ras effector and tumor suppressor, and its methylation has been found in a variety of tumor cell lines (including breast) and solid tumors (78–80), while the prognostic value of RASSF5 in breast cancer has hardly been reported. Taken together, apart from MARVELD2, the crucial roles of all the rest four genes in tumorigenesis have been demonstrated, and meanwhile large-scale multi-center clinical studies on these genes are needed because conflicting results often occur.

In the current study, since we haven’t found transcriptome data of CKI on TNBC patients or experimental animals in open-source databases so far, we used TNBC cell line samples with or without CKI perturbation for further analysis. This led to our findings cannot totally reflect on *in vivo* biological effects to some extent. Furthermore, we mainly applied the TNBC cohort with a relatively large sample size, RNA expression data and long-term survival information publicly accessed in the most authoritative breast cancer database METABRIC, so whether our results could be applied into the real-world population of TNBC patients should be further validated considering the high heterogeneity of TNBC. Ultimately, given that our findings came from comprehensive *in silico* analysis, additional evidence from larger TNBC cohorts with multi-omics profiles and long-term outcome information will be pivotal in supporting our results. In summary, our study integrates high throughput transcriptome data analysis and multiple machine learning methods for the first time to disclose the possible immune-related mechanisms and biomarkers of CKI on TNBC. Our findings may be useful in further studying the immune-based subtyping and prognostic biomarkers of TNBC, and our analytic methods would lay the foundation for further discovering possible therapeutic biomarkers of CKI on TNBC.

## Conclusions

In conclusion, this study proposes a predictive immunotherapy signature of CKI on TNBC, which would provide more evidence for survival prediction and treatment guidance on TNBC and a paradigm for exploring immunotherapy biomarkers of compound medicines. Meanwhile, further biological experiments and large-scale multi-center clinical studies are warranted to validate our findings since this study was conducted based on computational analysis.

## Data Availability Statement

The RNA-seq dataset of CKI on breast cancer MDA-MB-231 cells is publicly available at European Nucleotide Archive (ENA, http://www.ebi.ac.uk/ena) (35) with the accession number PRJNA517432 (36, 37) (BioProject: PRJNA517432, SRA: SRP182663, GEO: GSE125743). The METABRIC cohort (55) is publicly available at cBioPortal (http://www.cbioportal.org/). The GSE58812 dataset is publicly available at Gene Expression Omnibus (GEO, https://www.ncbi.nlm.nih.gov/geo/). The authors declare that all other data supporting the findings of our study are available within the paper.

## Author Contributions

XL conceived, designed and performed the research and wrote the paper. YW provided guidance in editing codes for bioinformatics analysis and substantive suggestions for revising the manuscript. YZhang provided useful suggestions in methodology. DB and YZhao provided computational resources for transcriptome data analysis. CW, SL, ZH, YS, FG, PY, CF, LS, JZ and HW provided suggestions for the manuscript. XD performed the UPLC-Q-TOF-MS experiment. XD and JW supervised the research. All authors contributed to the article and approved the submitted version.

## Funding

The study was financially supported by National Natural Science Foundation of China (Grant nos. 82074284).

## Conflict of Interest

The authors declare that the research was conducted in the absence of any commercial or financial relationships that could be construed as a potential conflict of interest.

## Publisher’s Note

All claims expressed in this article are solely those of the authors and do not necessarily represent those of their affiliated organizations, or those of the publisher, the editors and the reviewers. Any product that may be evaluated in this article, or claim that may be made by its manufacturer, is not guaranteed or endorsed by the publisher.

## Abbreviations

CKI, compound kushen injection; TNBC, triple-negative breast cancer; TME, tumor microenvironment; UPLC-Q-TOF-MS, ultra-performance liquid chromatography coupled to quadrupole time-of-flight mass spectrometry; RNA-seq, RNA sequencing; PCA, principal component analysis; DEGs, differential expression genes; GSVA, gene set variation analysis; GO, Gene Ontology; KEGG, Kyoto Encyclopedia of Genes and Genomes; DSS, disease specific survival; OS, overall survival; MFS, metastasis-free survival; ssGSEA, single sample gene set enrichment analysis; NES, normalized enrichment score; LASSO, least absolute shrinkage and selection operator; DCs, dendritic cells; NK cells, natural killer cells; MDSCs, myeloid-derived suppressor cells; NKT cells, natural killer T cells; pDCs, plasmacytoid dendritic cells; Tcm cells, central memory T cells; Tem cells, effector memory T cells; γδ T cells, gamma delta T cells; Treg cells, regulatory T cells; Tfh cells, T follicular helper cells; Th1 cells, type 1 T helper cells; Th2 cells, type 2 T helper cells; Th17 cells, type 17 T helper cells.
